# Wastewater-based epidemiology: the crucial role of viral shedding dynamics in small communities

**DOI:** 10.3389/fpubh.2023.1141837

**Published:** 2023-08-02

**Authors:** Marc-Denis Rioux, François Guillemette, Karine Lemarchand, Kim Doiron, Jean-François Lemay, Thomas Maere, Patrick Dolcé, Patrik Quessy, Nanouk Abonnenc, Peter A. Vanrolleghem, Dominic Frigon

**Affiliations:** ^1^Department of Mathematics and Engineering, Université du Québec à Rimouski, Quebec, QC, Canada; ^2^Department of Environmental Science, Université du Québec à Trois-Rivière, Quebec, QC, Canada; ^3^Institut des Sciences de la Mer, Université du Québec à Rimouski, Quebec, QC, Canada; ^4^Northern Institute for Research in Environment and Occupational Health and Safety, Quebec, QC, Canada; ^5^Centre National en Électrochimie et Technologies Environnementales, Cegep of Shawinigan, Quebec, QC, Canada; ^6^modelEAU, Département de génie civil et de génie des eaux, Université Laval, Quebec, QC, Canada; ^7^Centre Intégré de Santé et de services sociaux du Bas-Saint-Laurent, Quebec, QC, Canada; ^8^Department of Civil Engineering, McGill University, Quebec, QC, Canada

**Keywords:** wastewater-based epidemiology, viral shedding dynamics, small communities, wastewater surveillance, pathogens

## Abstract

**Background:**

Wastewater surveillance (WWS) of pathogens is a rapidly evolving field owing to the 2019 coronavirus disease pandemic, which brought about a paradigm shift in public health authorities for the management of pathogen outbreaks. However, the interpretation of WWS in terms of clinical cases remains a challenge, particularly in small communities where large variations in pathogen concentrations are routinely observed without a clear relation to clinical incident cases.

**Methods:**

Results are presented for WWS from six municipalities in the eastern part of Canada during the spring of 2021. We developed a numerical model based on viral kinetics reduction functions to consider both prevalent and incident cases to interpret the WWS data in light of the reported clinical cases in the six surveyed communities.

**Results:**

The use of the proposed numerical model with a viral kinetics reduction function drastically increased the interpretability of the WWS data in terms of the clinical cases reported for the surveyed community. In line with our working hypothesis, the effects of viral kinetics reduction modeling were more important in small communities than in larger communities. In all but one of the community cases (where it had no effect), the use of the proposed numerical model led to a change from a +1.5% (for the larger urban center, Quebec City) to a +48.8% increase in the case of a smaller community (Drummondville).

**Conclusion:**

Consideration of prevalent and incident cases through the proposed numerical model increases the correlation between clinical cases and WWS data. This is particularly the case in small communities. Because the proposed model is based on a biological mechanism, we believe it is an inherent part of any wastewater system and, hence, that it should be used in any WWS analysis where the aim is to relate WWS measurement to clinical cases.

## 1. Introduction

The main objective of the epidemiology of infectious diseases is to assess the distribution and effects of the etiologic agents on the health and wellbeing of human populations. In the context of the 2019 coronavirus disease (COVID-19) pandemic, this has led to large-scale testing of suspected infected individuals who underwent nasal and/or throat swab sampling followed by polymerase chain reaction (PCR) assay detection of severe acute respiratory syndrome coronavirus 2 (SARS-CoV-2). The objectives of these campaigns were 2-fold: (i) to identify positive virion carriers and (ii) to evaluate the acute progression of the pandemic in the population. These data are fundamental for predicting short-term demands on the healthcare system and assisting in the pandemic management decision-making process. However, the 2 years of the pandemic have shown that obtaining population data through individual tests is expensive both in terms of human and monetary resources.

An alternative approach based on wastewater surveillance (WWS) (1, 2) was proposed in the early phase of the COVID-19 pandemic (3). WWS provides two types of information. The first is the absence–presence in the community, which makes WWS a tool corollary to a canary in coal mines. The second type of information is the trend in the number of infections in the population, which has the potential to detect trends earlier than by monitoring clinical manifestations.

When coupled with a Geo-Data software (e.g., ArcGiS), WWS allows us for the first time to obtain on a daily basis *real population health data*. It is likely the first epidemiological data that are truly populational in nature. This reality needs to be emphasized as this new approach to generating population health data is an open field of research that did not previously exist. Classical epidemiological data are obtained from individuals presenting with clinical manifestations of infection with or without confirmatory molecular testing. Hence, classical data provide two specific types of information: positivity and identity of the carrier. In the case of WWS, information is populational. This means that one obtains information on the presence and abundance of an etiologic agent in a population of interest but not on the identity of the carriers. Thus, the data provide an indication of the health of the community as a whole. This is especially important to consider in an outbreak, such as the COVID-19 pandemic, because the optimal exploitation of WWS population data in relation to public health intervention is not straightforward and requires a new paradigm.

Correct interpretation of WWS data remains challenging as viral concentrations can vary substantially from 1 day to the next (4). In addition to the inherent daily variability in the concentration of SARS-CoV-2 in wastewater, the midterm temporal dynamics differs between large (collecting wastewater from hundreds of 1,000's of individuals) and small (collecting wastewater from a few tens of 1,000's of individuals) sewer systems. Although the temporal trends of SARS-CoV-2 concentrations in large systems (urban centers) typically follow a wave-like pattern over several weeks consequent to local outbreaks (4), these trends exhibit rapid increases and decreases in small systems (towns and rural communities) (5). Similar observations are reported here for samples obtained from sewer systems of different sizes. These strong signal oscillations make it challenging to adequately interpret wastewater data in small communities. Is the increasing trend observed today a strong indication of an increased number of incident^1^ cases in screened populations? To be a useful early indicator of population viral infection, WWS data need to be interpreted in terms of an overall viral attack in the population, hence presenting as little unexplainable variability as possible. It is hypothesized that the variability in temporal trends originates from the different aspects of the system being analyzed. Two important contributing factors are (i) interhuman variability in viral excretion kinetics coupled with the size of the outbreaks and (ii) the structure of the sewer system under study (including the water residence time and accumulation of solids). Following this hypothesis, large systems cover large populations with a high number of incident and prevalent^2^ cases at any time and with long water and solid residence times. Together, these elements tend to smooth out the variation in SARS-CoV-2 concentrations. Conversely, small systems receive fecal discharges from only a small number of prevalent cases at any time and are characterized by short water residence times.

From a biological perspective, we would expect prevalent cases to excrete a dwindling quantity of virions down to a null value within a certain number of days following infection. Concentrations measured from WWS reflect the combination of several individuals at different stages of infection. From this work hypothesis, we assumed that considering prevalent cases using a reduction function to consider the evolution of virions in time would better correlate with WWS data than simply considering incident cases. Because this effect is more impactful in small sewer systems, we hypothesized that considering viral excretion kinetics would improve the interpretability of WWS data in small sewer systems.

To test our hypothesis, we aimed to differentiate the effects of incident and prevalent COVID-19 cases on temporal trends in SARS-CoV-2 concentrations observed in wastewater samples from wastewater treatment plant influents in large urban settings and small towns and rural communities. To this end, we built a numerical model that explicitly considers the evolution of viral excretion over time. The main novelty of this work is that it identifies key differences in the interpretation of WWS data from large (densely populated) and small (with low population density) sewer systems (where data are sparser than in large communities) (6) and quantifies the effects of viral excretion kinetics in different contexts.

## 2. Materials and methods

### 2.1. Sampling

Our study was conducted in six municipalities of different sizes between January and June 2021 (Table 1). The size of the communities included in our study ranged from 2,000 to > 540,000 citizens. Table 1 shows the type of raw wastewater samples and the frequency of sampling used in each municipality. Grab samples refer to samples where all volumes are collected at instant t. Composite samples refer to either samples where constant volumes are collected over the course of a certain time (Composite 24 h) or samples where the volume collected during a time interval varies depending on the instantaneous flow rate (Composite-Flow 24 h). The concentrations of SRAS-CoV-2 in grab samples typically show a good correlation with the ones in composite samples (7–10). However, grab sampling taken in mid to late mornings leads to higher day-to-day variability and often to higher concentrations than composite sampling (7–10). In most communities, direct measurement of the flow rate was not possible, and the value was estimated based on the pump power and time of usage.

**Table 1:** Municipalities and their specifics.

**Municipality**	**Population**		**Start month in 2021**	**Sampling frequency**	**Type^I^**	Area classification^**^	**Average flow (m3/d)**
	**In sewershed**	**Density (citizen/km** ^2^ **)**					
Québec City	542,300	1,210	February	Daily	1	Large urban	359,000
Rimouski	48,650	146	January	3/week	1	Rural area	30,600 (est.^*^)
Rivière-du-Loup	19,450	237	Mars	1/week	1–2	Rural area	15,250 (est.)
				3/week			
Drummondville	68,600	310	April	3/week	1	Rural area	61,700 (est.)
Saint-Alexandre-de-Kamouraska	2,050	18	April	3/week	1	Rural area	1,185
La Tuque	11,125	0.39	Mars	2/week	1	Rural area	6,010 (est.)

* Flow rate estimated based on pump power and usage.

^**I**^Type: 1 is composite sample (24 h), and 2 is grab (instantaneous) sample.

** Classification based on POPCTRs (21).

Generally, samples were either analyzed the day they were sampled or refrigerated (4°C) for no more than 2 days before analysis. Some specimens (4 January to 15 January) sampled at the beginning of the project before the laboratory equipment for analysis was ready were frozen. Two different temperatures were used for frozen specimen: −20 and −80°C. Although reported work shows no loss of signal within 58 days at either −20 or −75°C (11), Centers for Disease Control and Prevention standards suggested the use of < −70°C for the preservation of samples (12).

### 2.2. Molecular analyses

The laboratory analysis of the samples was performed in three steps. Filtration was performed to concentrate the organic materials on the filters. SARS-CoV-2 virions tend to agglomerate with organic material rather than to float in free water. Thus, the collection of such organic materials and their concentrations on a filter during the filtration phase is crucial. Each sample underwent the first treatment in duplicate with a volume of 100 mL (50 mL for Quebec City) and was stirred at 200 rpm for 30 min at room temperature. After stirring, the pH was adjusted to 4.0 ± 0.5, and magnesium chloride (final concentration, 25 mM) was added. Each sample was filtered on 0.2 μm mixed cellulose ester filters with 47 mm diameter and stored at −80°C until further analysis.

RNA was extracted using the Qiagen RNeasy PowerMicrobiome Extraction Kit (QIAGEN). Briefly, all sample filters were cut into eight pieces and placed in 1.5 mL centrifuge tubes. In each tube, 100 μL of bovine respiratory syncytial virus (BRSV) was added as external control marker, and a reference sample of BSRV was extracted simultaneously to obtain the recovery rate to validate the extraction process. The remaining samples were extracted according to the manufacturer's instructions.

A one-step reverse transcription quantitative PCR (RT-qPCR) approach was used to quantify SARS-CoV-2, pepper mild mottle virus (PMMoV), and BRSV gene markers in wastewater samples. All primers and probes used in this study are listed in Table 2. For all samples, amplification reaction mixtures (final volume, 20 μL) contained 5 μL template RNA, 10 μL of 2 × Luna^®^ Universal Probe One-Step RT-qPCR (BioLabs Inc., New England), 0.25 μM for each forward and reverse primer, 0.125 μM of probe, and 1 μL of RT enzyme mix. The thermal cycling protocol was as follows: 10 min at 55°C for RT denaturation and 5 min at 95°C for initial denaturation followed by 40 cycles of two steps consisting of 10 s at 95°C and 30 s at 60°C. All RT-qPCR analyses were performed in triplicate (duplicate in Quebec) and in multiplex mode using a real-time PCR apparatus. Calibration curves were generated using the 2019-nCoV_N_Positive Control provided by Integrated DNA Technologies. The internal marker was PMMoV (14) and the external marker was BRSV. SARS-CoV-2 concentrations (gc/mL) were calculated from the cycle threshold (Ct) values using a calibration curve. Ct values < 38 were considered positive for SARS-CoV-2.

**Table 2:** Sequences of primers and probes for the detection of SARS-CoV-2, PMMoV, and BRSV.

**Target**	**Primer/probe name**	**Primer/probe sequence**	**References**
SARS-CoV-2	Forward primer	GAC CCC AAA ATC AGC GAA AT	(12)
	Reverse primer	TCT GGT TAC TGC CAG TTG AAT CTG	
	Probe (FAM)	FAM ACC CCG CAT/ZEN/TAC GTT TGG TGG ACC IABkFQ	
BRSV	Forward primer	GCA ATG CTG CAG GAC TAG GTA TAA T	(13)
	Reverse primer	ACA CTG TAA TTG ATG ACC CCA TTC T	
	Probe (Cy5)	Cy5 ACC AAG ACT/ZEN/TGT ATG ATG CTG CCA AAG CA IABkFQ	
PMMoV	Forward primer	TAC TTC GGC GTT AGG CAA TCA G	(14)
	Reverse primer	TGA AAC CAG TAG CAG GAA ATC TAA C	
	Probe (HEX)	5HEXCA GCA GTT CZENT CTG ATG TGT GG3IABkFQ	

### 2.3. Mathematic modeling and statistical analyses

For each sample, external and internal markers were assessed for aberrant data. Mean Ct values were converted into concentrations (gene copies per volume) using SARS-CoV-2 standard curves.

Viral load excretion from affected individuals varies with time from a maximal value to a null value at time *t*. Hence, incident cases obtained from health authorities from populational screening were used along a kinematic reduction-viral load function to consider this evolution in time. The modeled data comprise our first dataset and are referred to in the rest of the work as the *modeled equivalent shedding cases*. These modeled equivalent shedding cases were subsequently compared with the second dataset composed of SARS-CoV-2 concentrations obtained from the wastewater sample analyses. The main objective is to establish whether it is possible to define a function between SARS-CoV-2 concentration data and modeled data using regression analysis, assuming that modeled cases represent the *real* prevalence of viral infection in the population (or a close approximation). If applicable, this function would theoretically allow the calculation of an approximate number of prevalent cases based on WWS data.

To define the relationship between the two datasets, we hypothesized that the evolution of viral load shedding over time was an important factor. We call this evolution of the time of the viral load the kinetics reduction-viral load function.

To define the kinetics reduction-viral load function, we sought clinical data from anal swab and/or fecal analyses, where viral concentrations were measured at different time intervals. Positive carriers carry higher viral loads from throat swabs at or just before the onset of symptoms and that viral loads recede monotonically, leading to a significant decline in infectiousness 8–9 days after symptoms (15). For anal swabs, data (16) indicate that the mean duration of SARS-CoV-2 shedding is 17.2 days in feces, although live viruses have not been reported beyond 9 days of illness. Cevik et al. (16) also reported studies that show that the viral shedding duration is positively associated with age and severity of illness and that asymptomatic SARS-CoV-2 infection is associated with significantly lower viral loads after the initial stages compared with symptomatic individuals (faster clearance), although the initial viral load might be similar in both asymptomatic and symptomatic individuals. Because little is known about the *C*_*t*_ threshold used and because virus isolation was not conducted in studies on feces, it is difficult to establish a clear comparison. Considering these facts, the following hypotheses were used in this study to construct a recursive curve for viral loads: (i) the maximum viral load is assumed to be on the day of symptom onset (assumed to be on the day of a positive screening test), and (ii) the viral load monotonically decreases from the maximum value to zero at a certain time *t* (Equation 1):


(1) $$\begin{array}{l} {\Gamma \left(t\right)}_{v}=\Gamma_{0}\left(1-\beta \left(t\right)\right) \end{array}$$

where Γ_0_ is the maximal viral load and β(*t*) is the function that correlates the viral concentration at time *t* with the value of Γ_0_. The shape and value of Φ_*t*_ = (1−β(*t*)) is dependent on assumption (ii), which translates to a specific shape for the kinetics reduction shape function (Φ(*t*)). Several candidate Φ_*t*_ functions were tested, and their relative efficiencies in relating the SARS-CoV-2 concentration to the modeled cases were evaluated using the squared residual approach (Equation 2):


(2) $$\begin{array}{l} r^{2}=1-\frac{SSR}{TSS} \end{array}$$

where *r*^2^ is the coefficient of determination, SSR is the sum of squares of the residuals, and TSS is the total sum of squares. Specifically, we tested different hypotheses on the decay intensity for Γ_0_ to recede to a null value. All shape functions assumed an exponential form with varying degrees of decay between days 4 and 9. The regressions are based on linear and polynomial functions (third order). The *r*^2^ value for each regression in each city, as presented in Table 1, was calculated to establish the efficiency of the proposed procedure. We have included Supplementary material describing the other curves. However, because it was statistically impossible to distinguish between each curve and because the aim of this study was to demonstrate the usefulness of viral kinetics modeling, we presented only the curve that best fitted our data, based on Equation 2, and applied it to all municipalities:


(3) $$\begin{array}{l} \Phi_{t}=1-\beta \left(t\right) \end{array}$$

(4) $$\begin{array}{l} \beta \left(t\right)=1-0,455\ln\left(t\right)\;:t\in \mathbb{N}\;|\;\left[0,9\right] \end{array}$$

The data obtained from Φ_*t*_ (Equation 3) are compared with incident cases, that is, for β(1) = 0, β(1+Δ*t*) = 1.

With an in-house built code using *TcL*, a matrix of incident (*d*_0_) and prevalent (*d*_*t*_) cases [**C**] by the day of sampling (d) was compiled. The 1-β(*t*) coefficient is then provided in the form of a column vector quantity {1-β} so that the modeled equivalent shedding cases for each day of the study are obtained in a vector form {ς(d)} using Equation 1:


(5) ς(d)=C(d,t) Φ(t)

This is a simple vector-matrix product. To illustrate this process, we considered five incident cases at d = 0. The following day (d = 1), four incident cases occurred, and the next day (d = 2), seven. In this example, the [C] matrix is a 3 × 3 matrix, with three rows for d = 1, 2, and 3, and with three columns representing the day *t* that is appropriate for the 1-β(*t*) coefficient, where β(*t*) is given in Table 3 and ς(*d*) obtained by matrix × vector multiplication resulting in the following values:


(6) $$\begin{array}{l} C=\left[\begin{array}{ccc} 5 & 0 & 0\\ 4 & 5 & 0\\ 7 & 4 & 5 \end{array}\right]\Phi \left(t\right)=\left\{\begin{array}{c} 1\\ 0.68\\ 0.5 \end{array}\right\}\varsigma \left(d\right)=\left[\begin{array}{ccc} 5 & 0 & 0\\ 4 & 5 & 0\\ 7 & 4 & 5 \end{array}\right]*\left\{\begin{array}{c} 1\\ 0.68\\ 0.5 \end{array}\right\}\\ \;=\left\{\begin{array}{c} 5\\ 7.4\\ 12.2 \end{array}\right\} \end{array}$$

This simple matrix × vector product provides a time-dependent cumulative contribution of all incident and prevalent cases to the evolution of the viral load excreted, which better relates to the actual WWS measurements. This allowed us to consider the effects of viral kinetics by multiplying each incident and prevalent case by an appropriate factor. These factors are dependent on the assumptions considered in defining the viral kinetics reduction function.

**Table 3:** Candidate shape function parameter values.

**Days from symptoms onset**	β	
	**Viral kinetic function**	**Incident**
1	0	0
2	0.315	1
3	0.5	1
4	0.631	1
5	0.732	1
6	0.815	1
7	0.885	1
8	0.946	1
9	1	1

**Figure 1:**
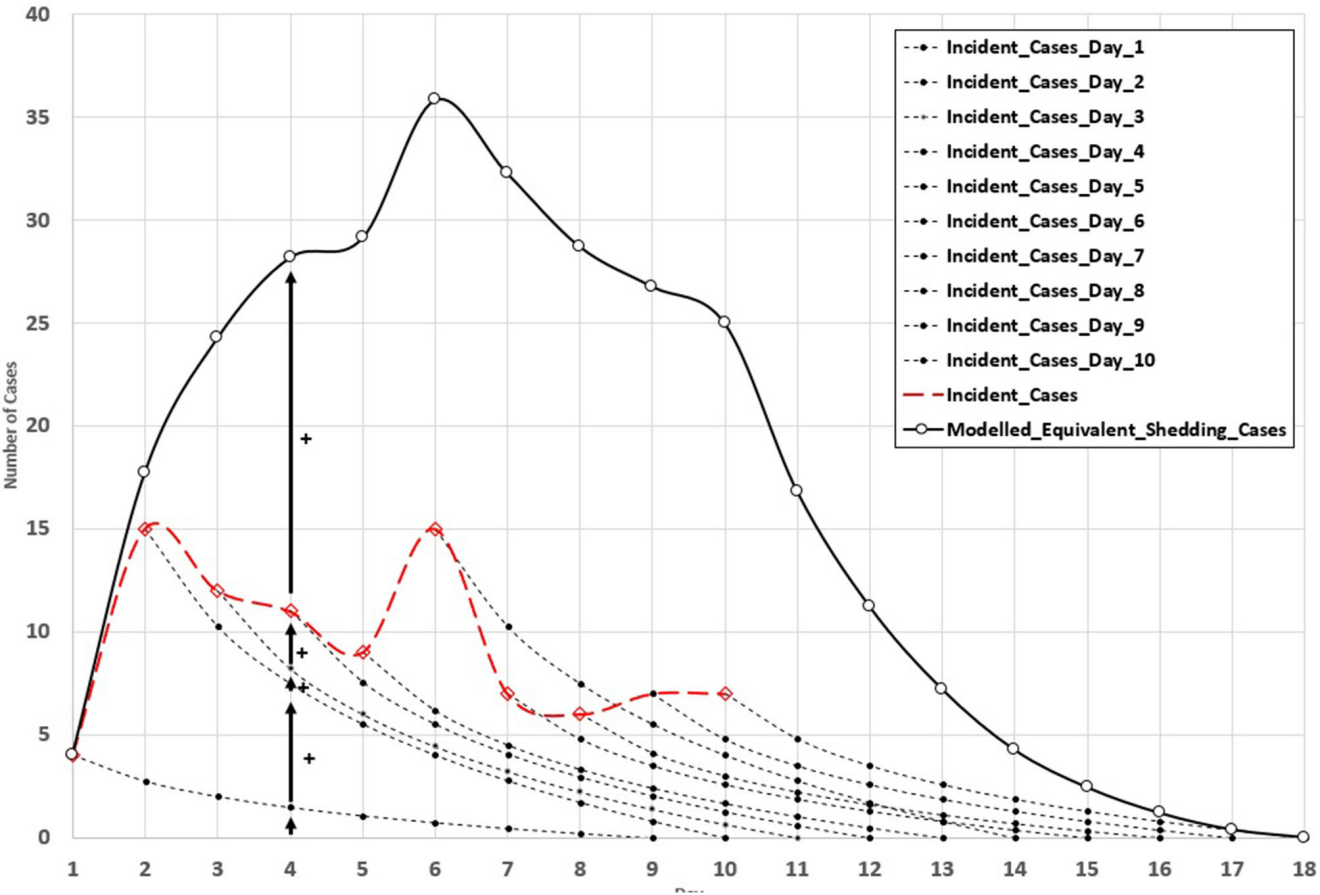
Effects of viral kinetics reduction on correlation with COVID 19 cases—Rimouski (more explanation, see text).

Figure 1 illustrates this mathematical approach. The horizontal axis in Figure 1 displays the day, whereas the vertical axis represents the number of clinical cases (or modeled equivalent shedding cases). In the example used, there were four incident cases on day 1. On day 2, there were 15 incident cases. Continuing with this logic would lead to the incident case curves (red dotted line) displayed in Figure 1. However, as previously stated, there is no logic behind the idea that incident cases from the previous day would stop contributing to the virion concentration measured in the WWS samples. Consider an example of the period between days 1 and 5. If we consider only incident cases, we would expect to observe a decrease in the trend of cases. However, from a wastewater concentration perspective, this would not be the case because prevalent cases still excrete virions in the sewers. Our proposed model, using shedding curves that decrease over time (dotted black lines, Figure 1), allows the effects of prevalent cases to be included in the modeled equivalent shedding cases (solid black curves, Figure 1).

For example, on day 4, the total number of modeled equivalent shedding cases is the sum of the contributions of all previously determined incident cases (days 1, 2, 3) and the incident cases on that day. This provides a significantly different portrait of what is happening, as can be observed by comparing the incident cases (dashed red line, Figure 1) with the modeled equivalent shedding cases (solid black line, Figure 1). This approach is underlined by a simple biological mechanism, that is, sick individuals keep excreting a receding number of virions during the course of the disease, from a peak at symptom onset to a null value after a certain amount of time. The work hypothesis was that the modeled equivalent shedding cases would be better related to the WWS concentration.

## 3. Results

**Figure 2:**
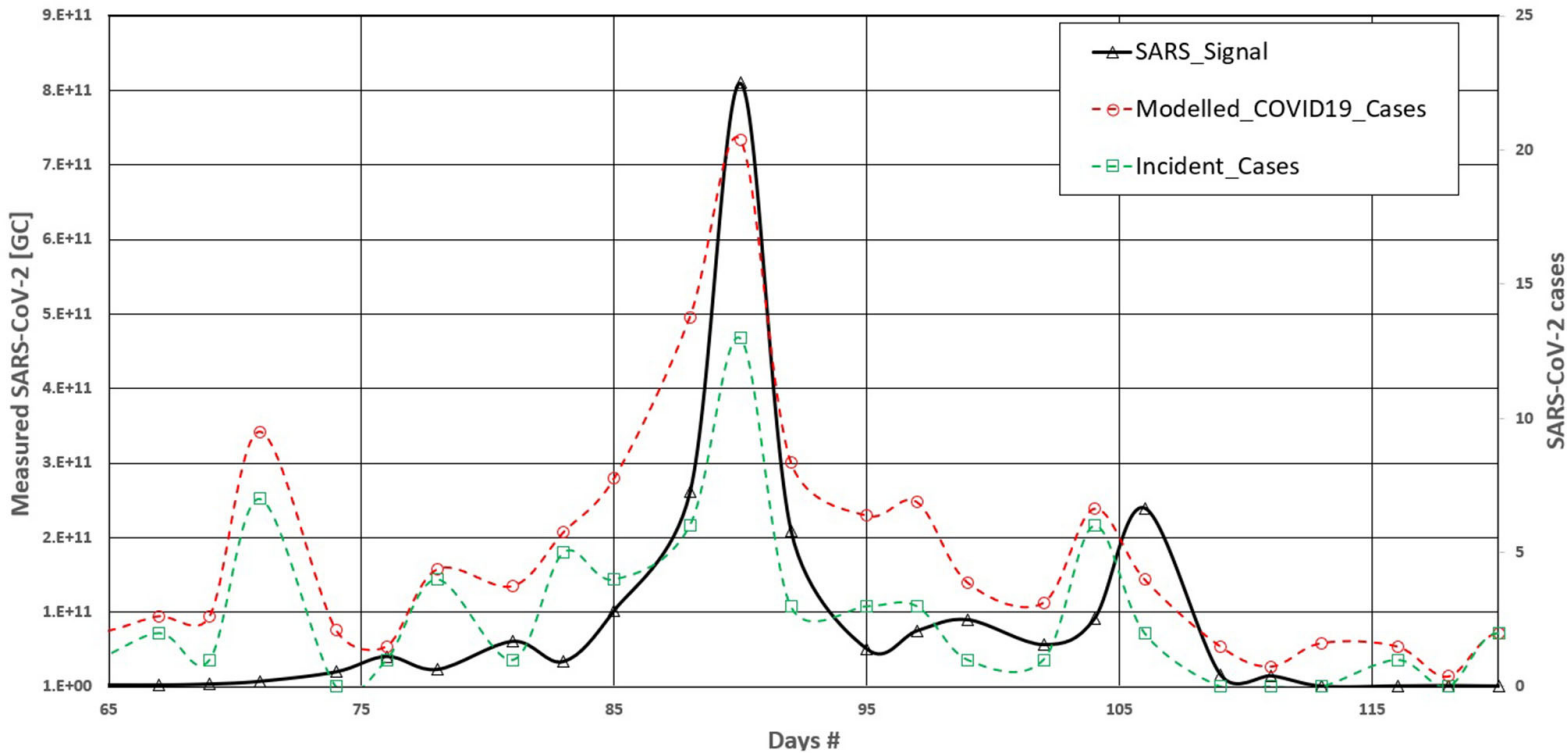
Effects of viral kinetics reduction on correlation with COVID 19 cases—Rimouski.

Figure 2 shows the curves of SARS-CoV-2 (Gc) in Rimouski City during the screening period (Table 1). The graph also displays the curves for incident cases. The incident case curve was highly jagged compared with the SARS-CoV-2 signal. Consequently, the relationship between the two datasets was not good, with an *r*^2^ of 0.61 using conventional linear regression. Figure 2 shows the modeled COVID-19 cases obtained using Equation 1. Using Equation 1 led to the smoothing of the curves by considering the prevalent cases and their evolution over time. The *r*^2^ between the modeled COVID-19 cases and the SARS-signal datasets significantly increased and reached a value of 0.69 for a standard linear regression. Using a third-order polynomial regression, *r*^2^ reached a value of 0.82, which was remarkably good given the uncertainty in the physical process being modeled.

There was a clear effect, for Rimouski city's dataset, when adding the effects of prevalent cases through viral kinetics reduction curves in the relationship between measured SARS-CoV-2 concentration in wastewater samples and populational COVID-19 cases. This is logical and follows a significantly simple biological mechanism; during the course of COVID-19 infection, the excretion of virions into the sewer network varies in time, with a maximum value at the beginning and dwindling down to a null value after a certain amount of time.

**Figure 3:**
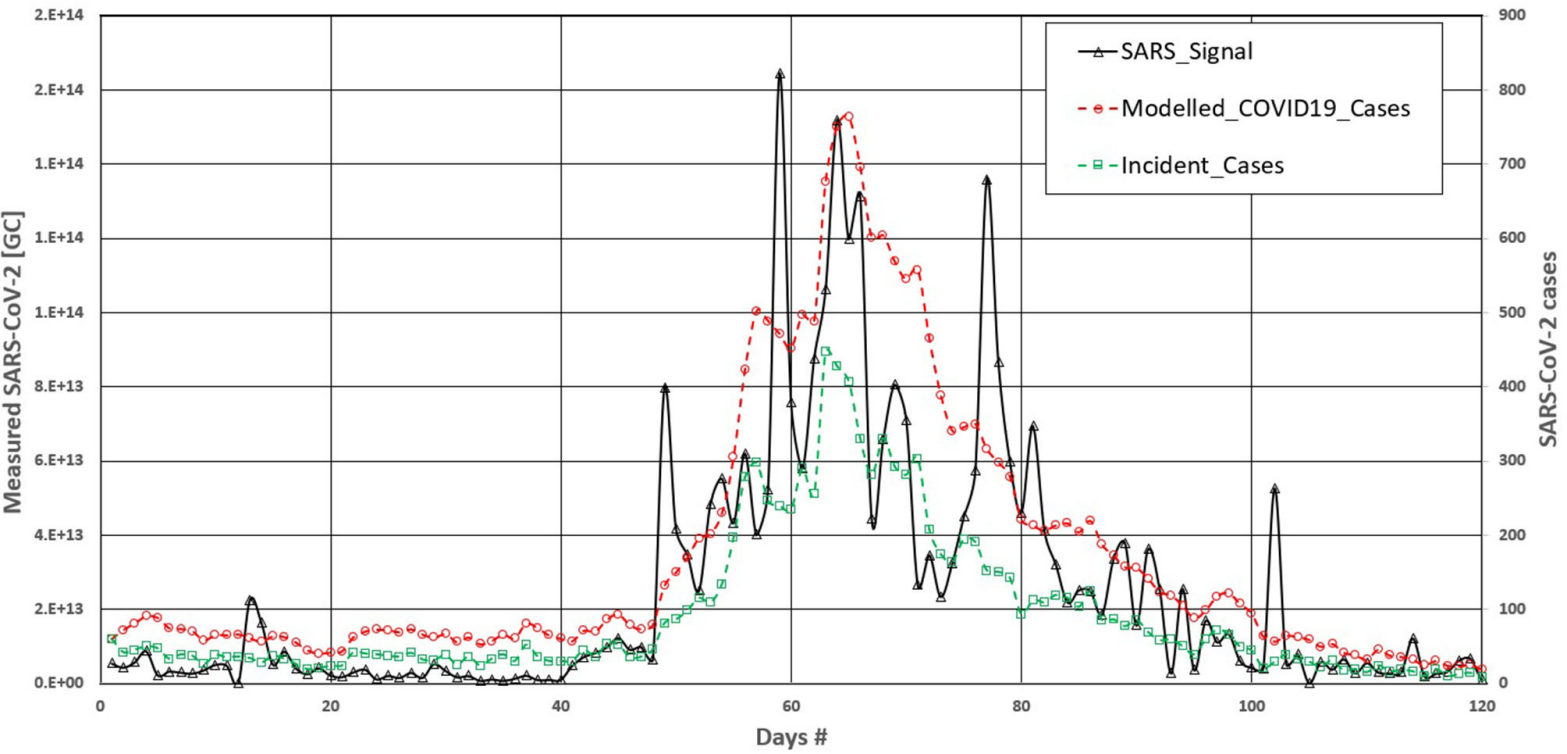
Effects of viral kinetics reduction on correlation with COVID 19 cases—Quebec.

In the case of larger communities, such as Quebec City, the picture is less clear. Figure 3 shows the evolution of SARS-CoV-2 signal in the wastewater samples from Quebec City. The graph also shows the values of the incident cases and the modeled COVID-19 cases (incident and prevalent) using the same viral kinetics reduction function as used by Rimouski.

The results in Figure 2 showed that using viral kinetics reduction led to the smoothing of the COVID-19 cases compared with the incident case curve. When considering the *r*^2^ value, using viral kinetics reduction led to a marginal decrease of *r*^2^, passing from 0.63 for the incident case dataset to 0.62 with the modeled datasets in standard linear regression and from 0.66 to 0.67 using the third order polynomial regression. We explain this by considering that the large number of incident cases reduces the effects of the prevalent cases in this population.

### 3.1. Population biomarkers

**Figure 4:**
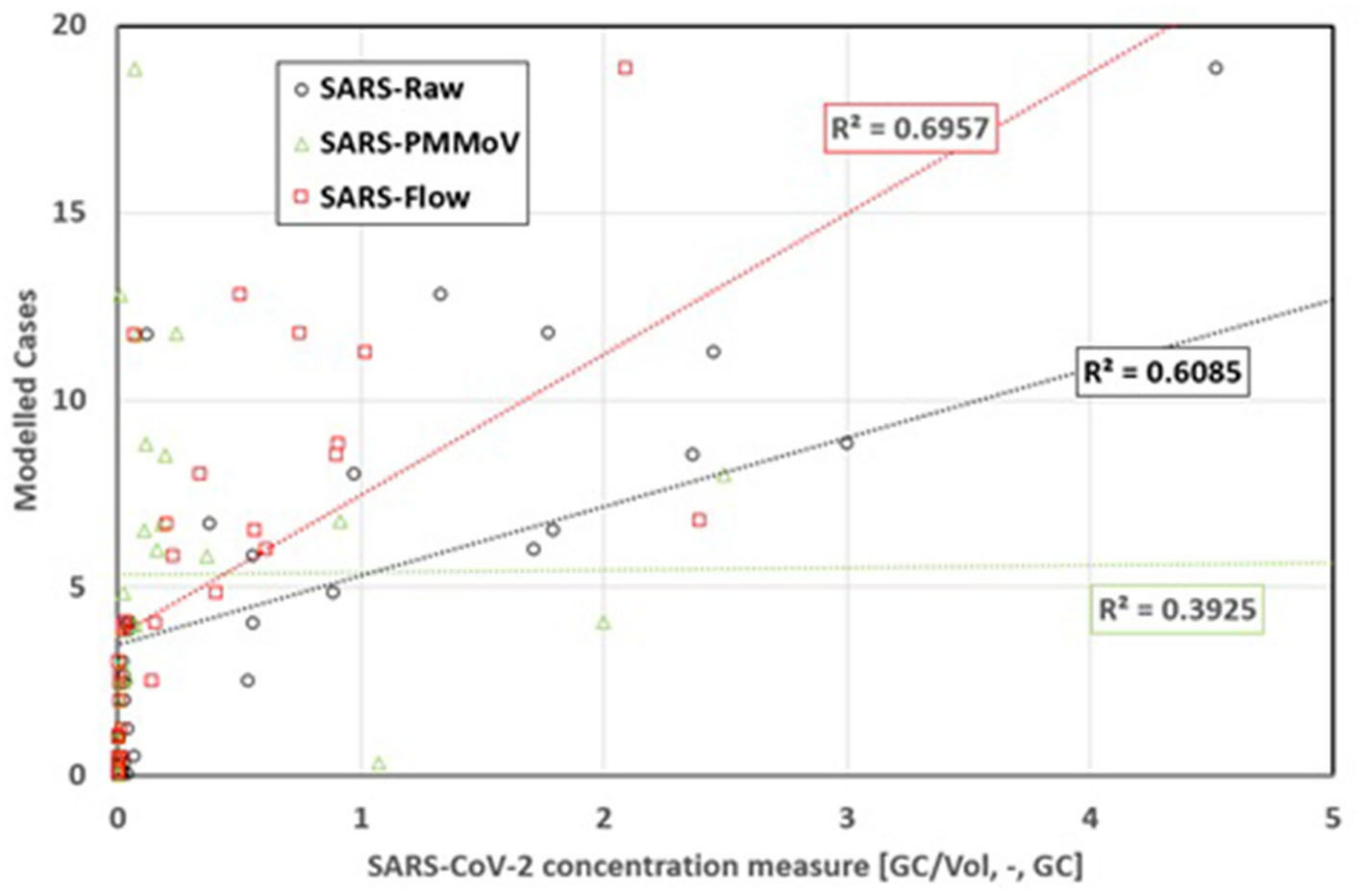
Comparison of the effect of pepper mild mottle virus and flow for normalization of data to modeled equivalent shedding cases—Rimouski.

Before presenting the complete results (see Section 3.2), it is important to detail how SARS-CoV-2 concentrations were normalized before being used in the regression. Based on previously published documentation available early during the pandemic (17, 18), PMMoV was initially considered to normalize the SARS-CoV-2 concentration. This biomarker is thought to be abundant in bell pepper-based foods, is unaffected by seasonal changes, and persists in wastewater (with a half-life of 6–10 days) from populated areas (19). Population biomarkers are important for two reasons. First, these biomarkers validate the presence of a sufficient quantity of organic materials in samples. Second, they can be used to normalize the concentration of detected virions to account for changes in wastewater dilution and differences in relative human waste input over time due to tourism, weekday commuters, and temporary workers (19). However, based on these data, PMMoV was a poor biomarker (Figure 4).

In fact, normalization of the SARS-CoV-2 concentration with PMMoV reduced the quality of the correlation obtained from the linear regression compared with the raw data. This result is in accordance with the recently published literature (19). The data indicate that flow rate is the most important factor related to virion concentration in the reported cases, and this conclusion is in line with that of a prior study (20).

### 3.2. Linear regression analysis results

As shown in Section 3, the data collected in spring 2021 showed a rapid increase and decrease when plotting COVID-19 incident cases vs. SARS-CoV-2 concentrations measured in wastewater samples obtained from small communities. Hence, in this study, we consider the effects of prevalent cases using a viral kinetics reduction function, as described previously. In this section, the results of the linear and polynomial regression analyses for all municipalities involved in the project are presented (Table 1).

Linear and third-order polynomial regression analyses were performed for each municipality. Graphs of SARS-CoV-2 concentration versus modeled equivalent shedding cases for all regression analysis are shown in Supplementary material.

Table 4 compares the *r*^2^ coefficients of all regressions for all communities considered between the datasets with incident cases only and those considering the modeled data.

**Table 4:** Summary of the regression analyses for all of the studied cities.

**Municipality**	***r*** ^**2**^			***r*** ^**2**^		
	**Linear incident**	**Linear kinetics reduction**	**Variation (%)**	**Poly (3rd) incident**	**Poly (3rd) kinetics reduction**	**Variation (%)**
Québec City	0.63	0.62	−1.6	0.66	0.67	+1.5
Rimouski	0.61	0.69	+13.1	0.6	0.82	+36.7
Rivière-du-Loup	0.7	0.69	−1.4	0.87	0.87	0.0
Drummondville	0.02	0.0054	−73.0	0.41	0.61	+48.8
Saint-Alexandre-de-Kamouraska	0.62	0.6	−3.2	0.88	0.91	+3.4
La Tuque	0.7	0.77	+10.0	0.63	0.83	+31.7

## 4. Discussion

Values of *r*^2^ considering both incident and prevalent cases using third-order polynomial regression along with a viral kinetics reduction function led to an increase in the correlation between the WWS data and clinical data (Table 4). This is particularly true in small communities. For urban centers with low population densities (Rimouski, Drummondville, and La Tuque), the modeled cases of COVID-19 were better correlated with SARS-CoV-2 concentrations measured in wastewater when prevalent cases were included according to the model. In the case of the city of Drummondville (+48.8%) WWS data were simply unusable without considering the viral kinetics evolution. In the case of a large community (Quebec, +1.5%), inclusion of viral kinetics had a less profound effect. This can be explained by the following mechanism.

### 4.1. Biological mechanism underlying the importance of viral kinetics

An individual's quantity of virions excreted in the feces varies during the course of SARS-CoV-2 infection. The maximum excretion of virions closely matches the initiation of disease symptoms and gradually decreases to a null value. When plotted in the time domain, variations in virion excretion can be described by a viral kinetics reduction function. During a viral outbreak, several individuals become ill at different times. Hence, the virion concentration in wastewater is a superposition of several individual viral kinetics reduction functions (Figure 1). It appears logical that as the number of infected individuals increases, this effect becomes less evident because of the cumulative effects of incident cases. However, when a limited number of individuals are affected, as is the case in small communities, failing to consider this effect may lead to a large discrepancy between the measured concentration in wastewater and clinical cases, making the interpretation of WWS data cumbersome.

To highlight this biological mechanism, we built a mathematical model that explicitly considers viral excretion kinetics. The main novelty of this work is that it identifies key differences in the interpretation of WWS data from large (densely populated) and small (with low population density) sewer systems and quantifies the effects of viral excretion kinetics in different contexts. Through regression analysis of SARS-CoV-2 measurements in wastewater samples from six municipalities located in Quebec (Canada), we showed that the inclusion of a viral kinetics reduction function to consider prevalent COVID-19 cases in the screened population led to an increase in correlation. The effect of this increase was especially visible in low-affected communities, where viral transmission remained low during SARS-CoV-2 screening. The impact was less evident in communities where a large number of incident cases concealed the effect of viral reduction.

### 4.2. Effects

Our results show that it is possible to accurately estimate prevalent cases at time t in a population using WWS data. However, making a good estimate requires the definition of a well-defined (data-supported) viral kinetics reduction function. Currently, the authors of this paper are unaware of any data that can provide specific viral kinetics reduction functions for the expression of SARS-CoV-2 virions in the feces of infected individuals in the general population. As stated previously, most supporting data originated from hospitalized individuals whose virion excretion might differ significantly from that of the general population. Furthermore, vaccination and varying viral lineages may produce different viral kinetics reduction functions. However, defining the maximum and minimum virion excretion evolutions in time based on a statistical analysis of data collected from voluntarily sick individuals representative of the general population would allow for a good estimation of population cases at any time based on WWS data. This would be revolutionary for WWS usage in epidemiology because WWS falls short when interpreting data. The ability to estimate population cases from WWS data using a sound biological mechanism would maximize the efficiency of WWS in future pandemic surveillance.

## 5. Conclusion

The data collected in this study support the hypothesis that the viral kinetics reduction function is a fundamental aspect of describing the biological evolution of SARS-CoV-2 virion shedding, which should be considered in the analysis. In all six municipalities studied, except for Rivière-du-Loup, the inclusion of such a reduction function led to an increase in the correlation for the third-order polynomial regression. For the specific cases of Rivière-du-Loup, we explained the negative effect of the nature of the COVID-19 infection in this particular community during our screening time. During our screening, the Rivière-du-Loup community observed two large outbreaks related to workers in a large company, but there was little contamination in the community. This means that, in both situations, we observed a large increase in incident cases on a daily basis over a relatively long period of time (≈*days*). In this context, the effect of the reduction in virions from the prevalent individual's excretion is lost in the increase in newly affected individuals.

This study aimed to differentiate the effects of incident and prevalent COVID-19 cases on the temporal trends in SARS-CoV-2 concentrations observed in wastewater samples from large urban settings and small towns and rural communities. Therefore, it was essential to consider the smoothing effect. There were more incidents in large cities than in small communities. In absolute terms, SARS-CoV-2 concentrations measured in wastewater were also higher in larger cities than in small communities. Because the incident cases are larger in number, the variation in SARS concentration is less significant from 1 day to the next because the kinetics of viral excretion is overwhelmed by the large number of incident cases, which contributes to the smoothing effect. Thus, the consideration of prevalent cases is important in small communities where fewer incident cases occur. When there are few incident cases in a population, the contribution of the prevalent cases to the SARS-CoV-2 signal is more significant.

However, our data suggest that the normalization of SARS-CoV-2 concentrations in wastewater samples should consider the flow rate and that there is a lack of consensus on a good biomarker for population normalization and a need for studies on this particular question.

The specific contributions of this work include (1) compelling evidence from several rural and urban municipalities to robustly demonstrate that viral kinetics-induced variability needs to be considered, especially in lower-density communities, and (2) a simple model to account for this viral kinetics effect and its application to regression analysis for estimating SARS-CoV-2 prevalence in screened populations.

## 6. Limitations and recommendations

Because the biological model proposed in this study was established based on the basic assumption of disease progression, it is thought to be general and applicable to various types of biological etiologic agents worldwide. However, the specific shape function used in the model varies, depending on several factors. For example, in the case of SARS-CoV-2, the vaccination status, age, and viral lineage are all susceptible to influence the function used in the model. It is expected that virus different from that of SARS-CoV-2 has different shape functions. Hence, our results should be understood in this context as a general demonstration of the importance of considering disease evolution in affected individuals in the interpretation of wastewater data while keeping in mind that the specific function developed in this study should not be directly used in another context.

These findings highlight the need for further studies on the temporal evolution of virion excretion in different pathogens, including different SARS-CoV-2 lineages. If wastewater data are used to estimate infection in a population, which should be the main objective of this technique, studies on the evolution of virion excretion in body fluids are fundamental to refining the reduction function used in our model and allowing such estimates to be made on a sound basis.

## Data availability statement

The datasets used for the regression analysis in this article can be found at https://zenodo.org/record/8010506.

## Author contributions

FG, J-FL, PQ, and NA substantially contributed to the article by providing data for Drummondville, Sainte-Alexandre-de-Kamouraska, and La Tuque and suggested ideas for the writing of the article. KL, KD, PV, and TM substantially contributed to the article by providing data for Rimouski, Quebec, and Rivière-du-Loup and suggested several important modifications to the article. M-DR contributed to the article by producing the numerical model, making the calculations, producing the image, writing the article, and maturing key ideas with the other authors of the paper. DF contributed to the article by reviewing key ideas, suggesting several critically important intellectual elements of the model, and rewriting several parts of the document. PD contributed to the work by providing key intellectual ideas and important data. All authors contributed to the article and approved the submitted version.

## Footnotes

^1^Individuals changing in status from non-disease to disease carrier (new cases).

^2^Individuals with positive disease over a specified period of time (existing cases).

## References

[1] Ahmed W Tscharke B Bertsch PM Bibby K Bivins A Choi P. SARS-CoV-2 RNA monitoring in wastewater as a potential early warning system for COVID-19 transmission in the community: a temporal case study. Sci Total Environ. (2021) 761:144216. 10.1016/j.scitotenv.2020.144216 33360129 PMC7718102

[2] Pöyry T Stenvik M Hovi T. Viruses in sewage waters during and after a poliomyelitis outbreak and subsequent nationwide oral poliovirus vaccination campaign in Finland. Appl Environ Microbiol. (1988) 54:371–4. 10.1128/aem.54.2.371-374.1988 2833160 PMC202459

[3] Lodder W de Roda Husman AM. SARS-CoV-2 in wastewater: potential health risk, but also data source. Lancet Gastroenterol Hepatol. (2020) 5:533–4. 10.1016/S2468-1253(20)30087-X 32246939 PMC7225404

[4] Nourbakhsh S Fazil A Li M Mangat CS Peterson SW Daigle J. A wastewater-based epidemic model for SARS-CoV-2 with application to three Canadian cities. medRxiv. (2021) 2021:21260773. 10.1101/2021.07.19.21260773 35462206 PMC8993419

[5] Daigle J Racher K Hazenberg J Yeoman A Hannah H Duong D. A sensitive and rapid wastewater test for SARS-CoV-2 and its use for the early detection of a cluster of cases in a remote community. Appl Environ Microbiol. (2022) 88:e0174021. 10.1128/aem.01740-21 34985977 PMC8904056

[6] Hubert CRJ Acosta N Waddell BJM Hasing ME Qiu Y Fuzzen M. Tracking emergence and spread of SARS-CoV-2 Omicron variant in large and small communities by wastewater monitoring in Alberta, Canada. Emerg Infect Dis. (2022) 28:1770–6. 10.3201/eid2809.220476 35867051 PMC9423933

[7] Kmush BL Monk D Green H Sachs DA Zeng T Larsen DA. Comparability of 24-hour composite and grab samples for detection of SARS-2-CoV RNA in wastewater. FEMS Microbes. (2022) 3:17. 10.1093/femsmc/xtac017 37332496 PMC10117866

[8] George AD Kaya D Layton BA Bailey K Mansell S Kelly C. Impact of sampling type, frequency, and scale of the collection system on SARS-CoV-2 quantification fidelity. Environ Sci Technol Lett. (2022) 9:160–5. 10.1021/acs.estlett.1c00882 37566370

[9] Augusto MR Claro ICM Siqueira AK Sousa GS Caldereiro CR Duran AFA. Sampling strategies for wastewater surveillance: evaluating the variability of SARS-CoV-2 RNA concentration in composite and grab samples. J Environ Chem Eng. (2022) 10:107478. 10.1016/j.jece.2022.107478 35251931 PMC8882035

[10] Bivins A North D Wu Z Shaffer M Ahmed W Bibby K. Within- and between-day variability of SARS-CoV-2 RNA in municipal wastewater during periods of varying COVID-19 prevalence and positivity. ACS ES&T Water. (2021) 1:2097–108. 10.1021/acsestwater.1c00178

[11] Hokajärvi A-M Rytkönen A Tiwari A Kauppinen A Oikarinen S Lehto K-M. The detection and stability of the SARS-CoV-2 RNA biomarkers in wastewater influent in Helsinki, Finland. medRxiv. (2020) 2020:20234039. 10.1101/2020.11.18.20234039 33513496 PMC7825999

[12] Centers for Disease Control and Prevention (CDC) National Wastewater Surveillance System (NWSS). Developing a Wastewater Surveillance Sampling Strategy. (2020).

[13] Boxus M Letellier C Kerkhofs P. Real Time RT-PCR for the detection and quantitation of bovine respirtory syncytial virus. J Virol Methods. (2005) 125:125–30. 10.1016/j.jviromet.2005.01.008 15794981

[14] Kitajima M Sassi HP Torrey JR. Pepper mild mottle virus as a water quality indicator. NPJ Clean Water. (2018) 1:19. 10.1038/s41545-018-0019-536851496

[15] He X Lau EHY Wu P Deng X Wang J Hao X. Temporal dynamics in viral shedding and transmissibility of COVID-19. Nat Med. (2020) 26:672–5. 10.1038/s41591-020-0869-5 32296168

[16] Cevik M Tate M Lloyd O Maraolo AE Schafers J Ho A. SARS-CoV-2, SARS-CoV, and MERS-CoV viral load dynamics, duration of viral shedding, and infectiousness: a systematic review and meta-analysis. Lancet Microbe. (2021) 2:e13–22. 10.1016/S2666-5247(20)30172-5 33521734 PMC7837230

[17] Rosario K Symonds EM Sinigalliano C Stewart J Breitbart M. Pepper mild mottle virus as an indicator of fecal pollution. Appl Environ Microbiol. (2009) 75:7261–7. 10.1128/AEM.00410-09 19767474 PMC2786529

[18] Kitajima M Iker BC Pepper IL Gerba CP. Relative abundance and treatment reduction of viruses during wastewater treatment processes–identification of potential viral indicators. Sci Total Environ. (2014) 488–9:290–6. 10.1016/j.scitotenv.2014.04.087 24836386

[19] Hsu SY Bayati MB Li C Hsieh HY Belenchia A Klutts J. Biomarkers selection for population normalization in SARS-CoV-2 wastewater-based epidemiology. medRxiv. (2022) 2022:22272359. 10.1101/2022.03.14.22272359 36030667 PMC9376872

[20] Isaksson F Lundy L Hedström A Székely AJ Mohamed N. Evaluating the use of alternative normalization approaches on SARS-CoV-2 concentrations in wastewater: experiences from two catchments in northern sweden. Environments. (2022) 9:39. 10.3390/environments9030039

[21] Canada Go. Population Centre and Rural Area Classification. Stats Can: 2016 06-09-2022. (2017).

