# Supplementary material for: Wastewater-based epidemiology: the crucial role of viral shedding dynamics in small communities
Source: Front Public Health. 2023 Aug 2;11:1141837. doi: 10.3389/fpubh.2023.1141837 (PMC10433918; doi:10.3389/fpubh.2023.1141837)
Supplement: Supplementary file 1 [file Data_Sheet_1.zip › Supplementary Material Description.docx]

Supplementary Material

Wastewater epidemiology: The crucial role of viral shedding dynamics in small communities

Marc-Denis Rioux^1^, François Guillemette^2^, Karine Lemarchand^3^, Kim Doiron^4^, Jean-François Lemay^5^, Thomas Maere^6^, Patrick Dolce^7^, Patrik Quessy^5^, Nanouk Abonnenc^5^, Peter A. Vanrolleghem^6^, Dominic Frigon^8^

**Correspondence:** Marc-Denis Rioux: marc-denis_rioux@uqar.ca

# Supplementary material

The supplementary material includes two sets of graphs: 1) linear and polynomial regression for all cities listed in the present study (Figures 5 to 16) and 2) a comparison between the two distinct viral kinetics reduction functions, incident cases and the rolling 7-day sum for Quebec City, Rimouski, Rivière-du-Loup, Drummondville and La Tuque (Figures 17 to 21).

Data comparison between distinct viral kinetic reduction functions were not included in the main article because it was deemed impossible to statistically establish the “good” kinetics function. This study aimed to demonstrate that the model increases the accuracy between clinical cases and wastewater surveillance (WWS) data. Therefore, the best-fit curves were selected. We again emphasis here that our proposed model is based on a natural biological mechanism but that the specific forms of the viral kinetic reduction functions need to be better defined. Our proposed mechanism is general enough to be applicable to others pathogens thought the specific shape of the kinetic reduction functions would likely vary and need to be defined depending on several parameters.

As shown in Figures 17 to 21 below, it is difficult to identify a single curve that best fits all cities. Notably, for Drummondville and La Tuque, the 7-day rolling sum produced a better correlation than the proposed model. However, the gain is relatively small in both cases, and the 7-day rolling sum seems to produce the worst results in all other cities, especially in cities where large amounts of WWS data are available (Rimouski and Quebec). This confirms our hypothesis that considering prevalent cases is fundamental, but the best way of doing it still requires studies.

**Figure Legends**

Figure 5: Linear regression between concentration (GC/day) and modeled equivalent shedding cases for Rimouski - Orange dots, incident cases; green dots, prevalent + incident cases

Figure 6: Third-order polynomial regression between SARS-CoV-2 concentration (GC/day) and modeled equivalent shedding cases for Rimouski - Orange dots, incident cases; black dots, prevalent + incident cases

Figure 7: Linear regression between SARS-CoV-2 concentration (GC/day) and modeled equivalent shedding cases for Quebec City - Orange dots, incident cases; green dots, prevalent + incident cases

Figure 8: Third-order polynomial regression between SARS-CoV-2 concentration (GC/day) and modeled equivalent shedding cases for Quebec City - Orange dots, incident cases; black dots, prevalent + incident cases

Figure 9: Linear regression between SARS-CoV-2 concentration (GC/day) and modeled equivalent shedding cases for Drummondville - Orange dots, incident cases; green dots, prevalent + incident cases

Figure 10: Third-order polynomial regression between SARS-CoV-2 concentration (GC/day) and modeled equivalent shedding cases for Drummondville - Orange dots, incident cases; black dots, prevalent + incident cases

Figure 11: Linear regression between SARS-CoV-2 concentration (GC/day) and modeled equivalent shedding cases for Rivière-du-Loup- Orange dots, incident cases; green dots, prevalent + incident cases

Figure 12: Third-order polynomial regression between SARS-CoV-2 concentration (GC/day) and modeled equivalent shedding cases for Rivière-du-Loup - Orange dots, incident cases; black dots, prevalent + incident cases

Figure 13: Linear regression between SARS-CoV-2 concentration (GC/day) and modeled equivalent shedding cases for Saint-Alexandre-de-Kamouraska - Orange dots, incident cases; green dots, prevalent + incident cases

Figure 14: Third-order polynomial regression between SARS-CoV-2 concentration (GC/day) and modeled equivalent shedding cases for Saint-Alexandre-de-Kamouraska - Orange dots, incident cases; black dots, prevalent + incident cases

Figure 15: Linear regression between SARS-CoV-2 concentration (GC/day) and modeled equivalent shedding cases for La Tuque - Orange dots, incident cases; green dots, prevalent + incident cases

Figure 16: Third-order polynomial regression between SARS-CoV-2 concentration (GC/day) and modeled equivalent shedding cases for La Tuque - Orange dots, incident cases; black dots, prevalent + incident cases

Figure 17: Third-order polynomial regression between modeled equivalent shedding cases and SARS-CoV-2 concentration (log) for Rimouski - Black dot, function A; red dots, function B; blue dots, function C; orange dots, 7-day rolling sums

Figure 18: Third-order polynomial regression between modeled equivalent shedding cases and SARS-CoV-2 concentration (log) for Quebec - Black dot, function A; red dots, function B; blue dots, function C; orange dots, 7-day rolling sums

Figure 19: Third-order polynomial regression between modeled equivalent shedding cases and SARS-CoV-2 concentration (log) for La Tuque - Black dot, function A; red dots, function B; blue dots, function C; orange dots, 7-day rolling sums

Figure 20: Third-order polynomial regression between modeled equivalent shedding cases and SARS-CoV-2 concentration (log) for Drummondville - Black dot, function A; red dots, function B; blue dots, function C; orange dots, 7-day rolling sums

Figure 21: Third-order polynomial regression between modeled equivalent shedding cases and SARS-CoV-2 concentration (log) for Rivière-Du-Loup - Black dot, function A; red dots, function B; blue dots, function C; orange dots, 7-day rolling sums
