# Supplementary material for: Wastewater-based epidemiology: the crucial role of viral shedding dynamics in small communities
Source: Front Public Health. 2023 Aug 2;11:1141837. doi: 10.3389/fpubh.2023.1141837 (PMC10433918; doi:10.3389/fpubh.2023.1141837)
Supplement: Supplementary file 1 [file Data_Sheet_1.zip › Table 5.docx]

Table 5: Shape function parameter values used to define the viral kinetics reduction function

| **Days from** | $\beta$ | | | |
| --- | --- | --- | --- | --- |
| **symptom onset** | **A** | **B** | **C** | **D** |
| 1 | 0 | 0 | 0 | 0 |
| 2 | 0.5 | 0.315 | 1 | 0 |
| 3 | 0.75 | 0.5 | 1 | 0 |
| 4 | 0.875 | 0.631 | 1 | 0 |
| 5 | 1 | 0.732 | 1 | 0 |
| 6 | 1 | 0.815 | 1 | 0 |
| 7 | 1 | 0.885 | 1 | 0 |
| 8 | 1 | 0.946 | 1 | 1 |
| 9 | 1 | 1 | 1 | 1 |
