# Supplementary figures and images for: Wastewater-based epidemiology: the crucial role of viral shedding dynamics in small communities
Source: Front Public Health. 2023 Aug 2;11:1141837. doi: 10.3389/fpubh.2023.1141837 (PMC10433918; doi:10.3389/fpubh.2023.1141837)

Figure 8


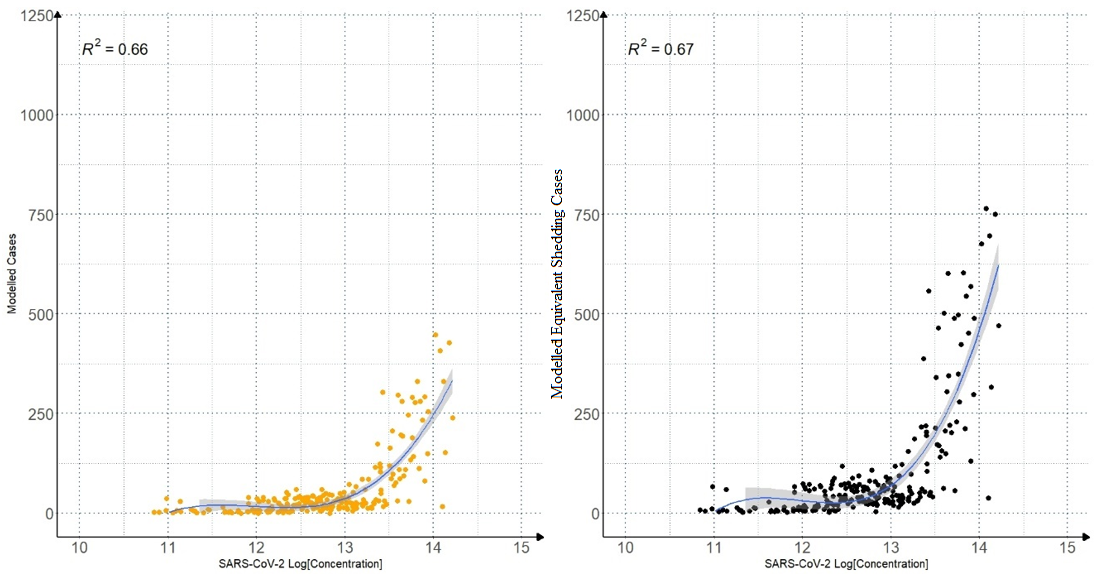

Supplement: Supplementary file 1 [file Data_Sheet_1.zip › Frontier_Epidemiology_English_EditingVersion_Figure8.docx]

Figure 9


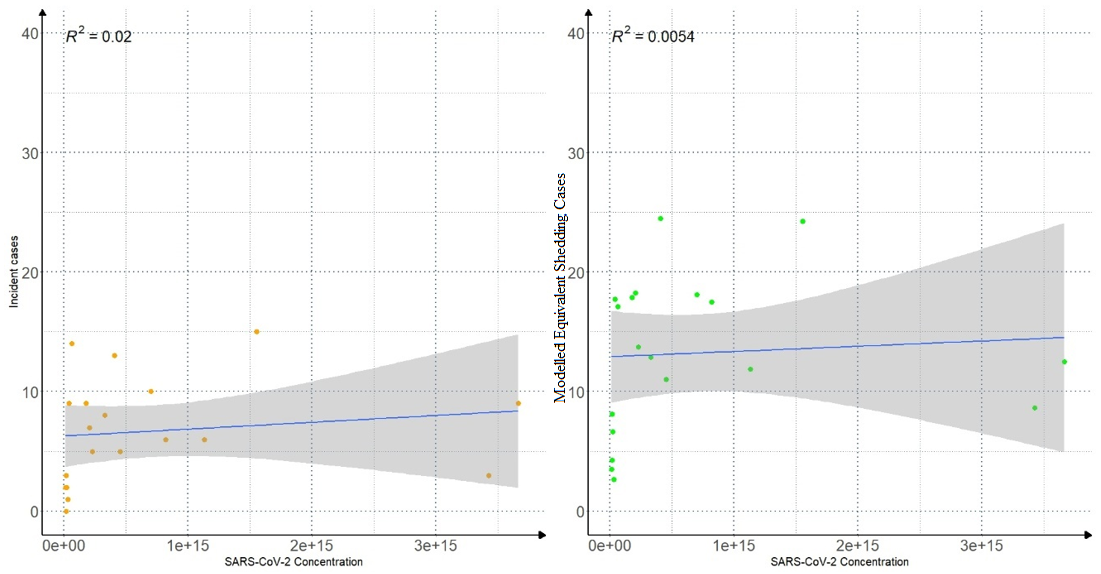

Supplement: Supplementary file 1 [file Data_Sheet_1.zip › Frontier_Epidemiology_English_EditingVersion_Figure9.docx]

Figure 10


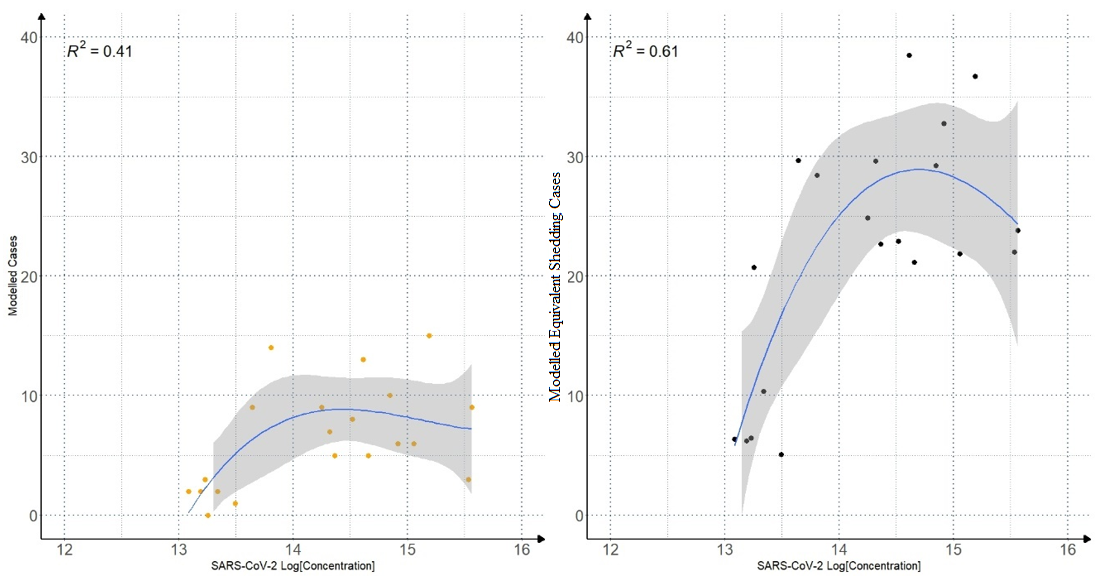

Supplement: Supplementary file 1 [file Data_Sheet_1.zip › Frontier_Epidemiology_English_EditingVersion_Figure10.docx]

Figure 11


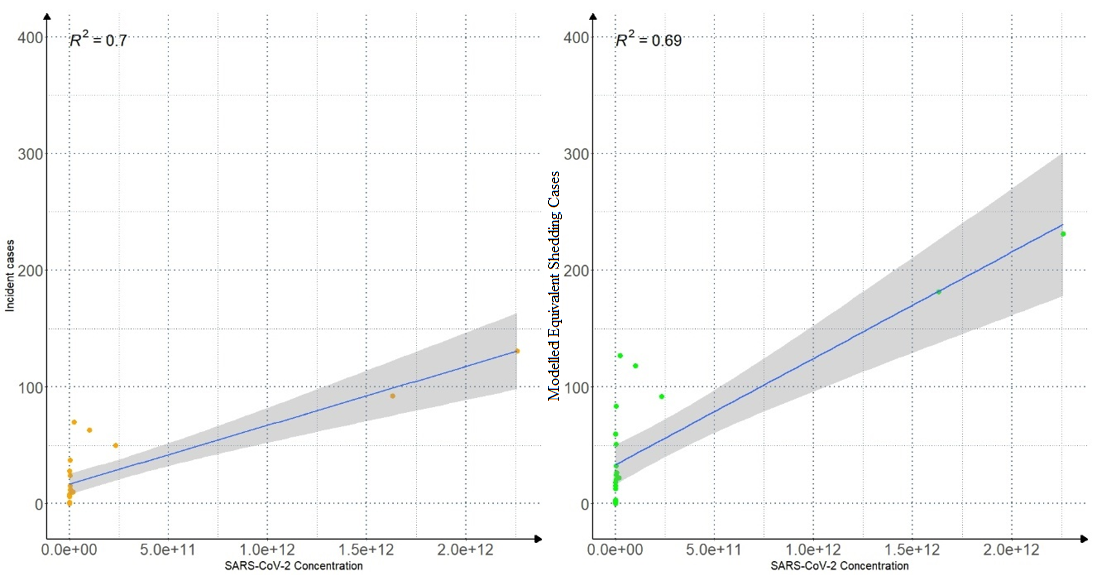

Supplement: Supplementary file 1 [file Data_Sheet_1.zip › Frontier_Epidemiology_English_EditingVersion_Figure11.docx]

Figure 12


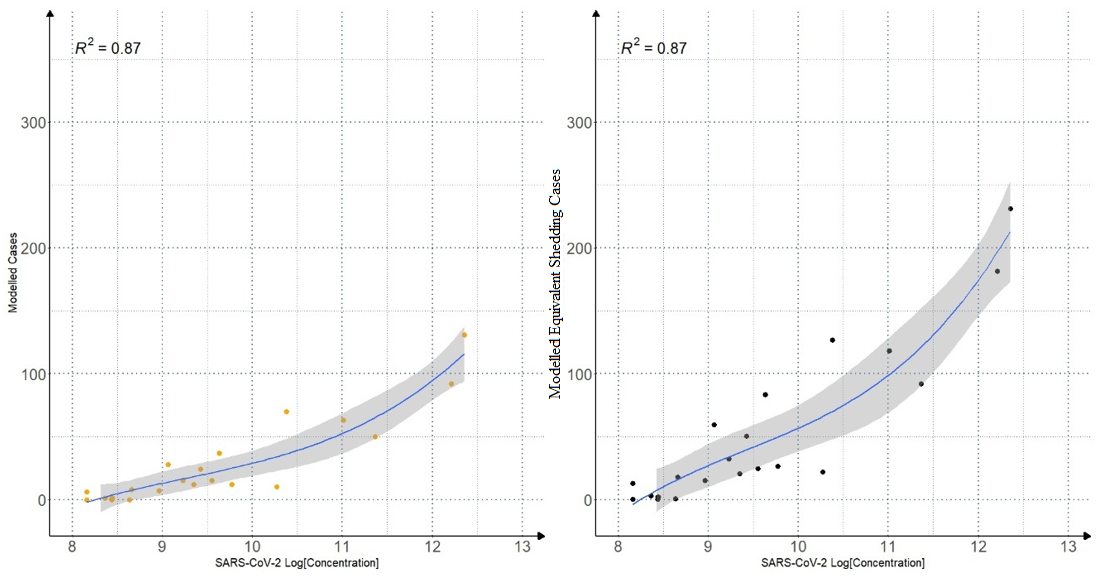

Supplement: Supplementary file 1 [file Data_Sheet_1.zip › Frontier_Epidemiology_English_EditingVersion_Figure12.docx]

Figure 13


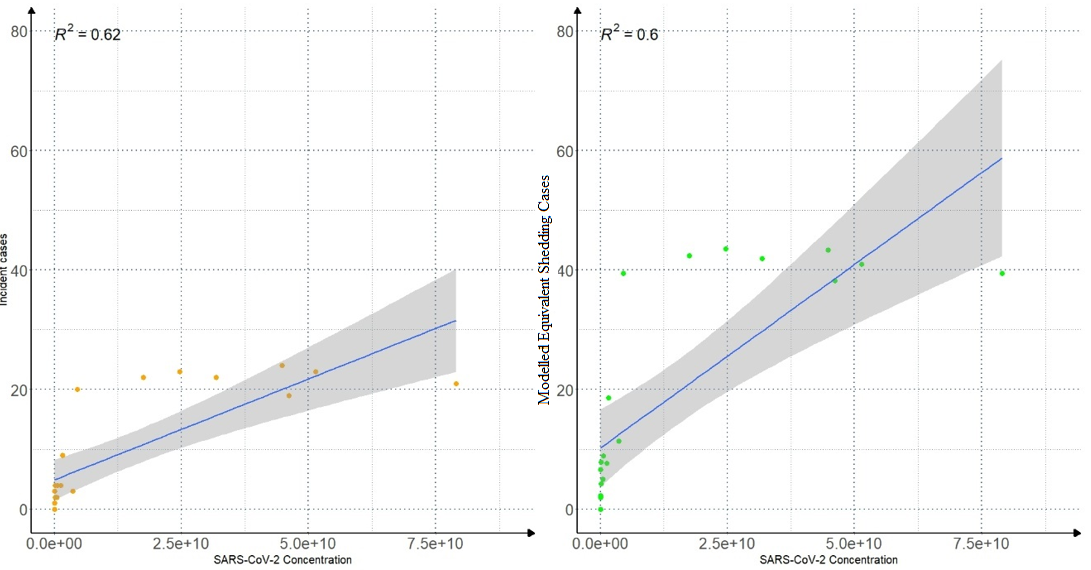

Supplement: Supplementary file 1 [file Data_Sheet_1.zip › Frontier_Epidemiology_English_EditingVersion_Figure13.docx]

Figure 14


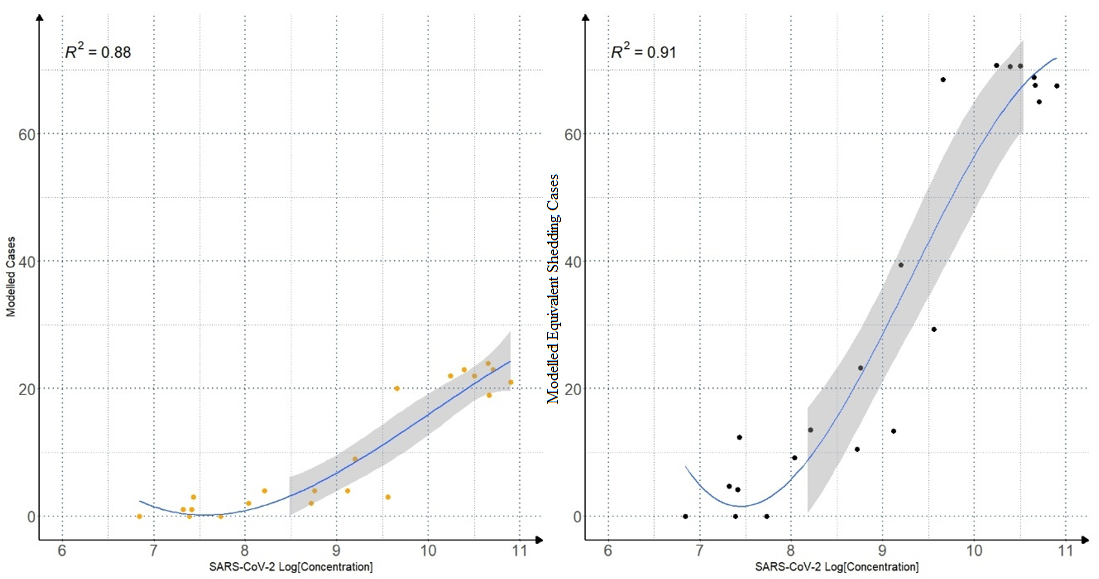

Supplement: Supplementary file 1 [file Data_Sheet_1.zip › Frontier_Epidemiology_English_EditingVersion_Figure14.docx]

Figure 15


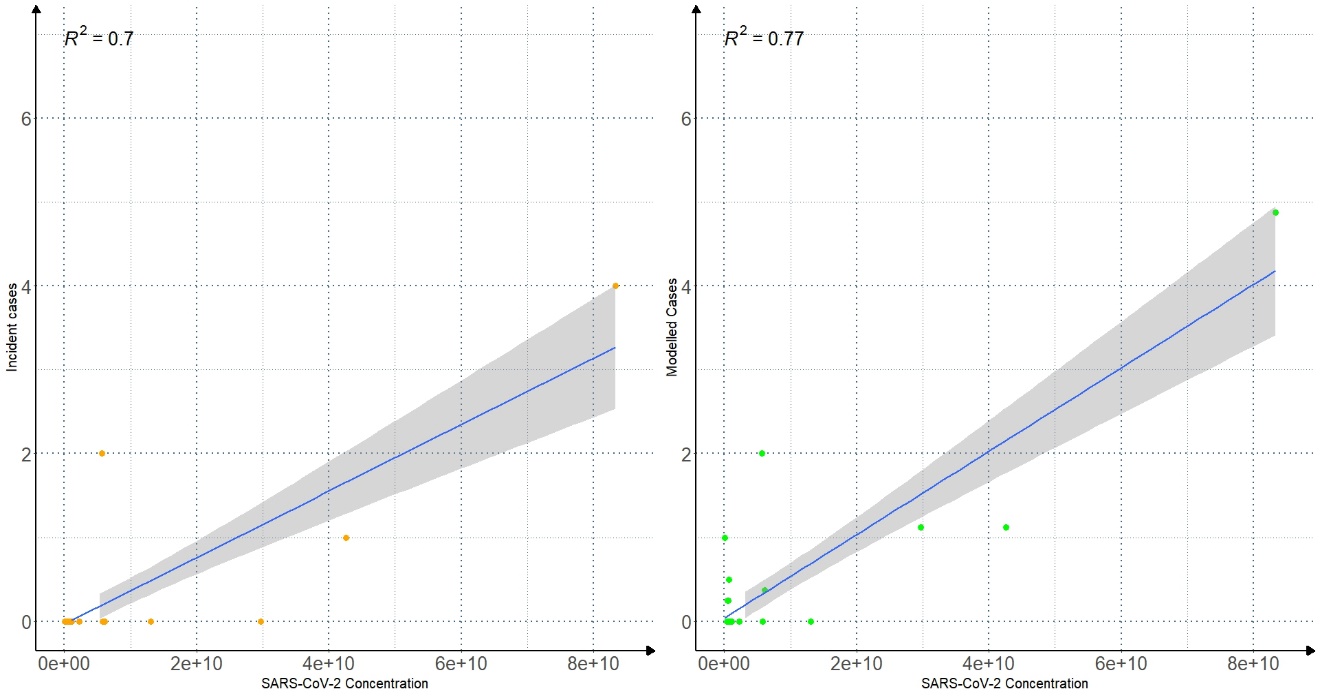

Supplement: Supplementary file 1 [file Data_Sheet_1.zip › Frontier_Epidemiology_English_EditingVersion_Figure15.docx]

Figure 16


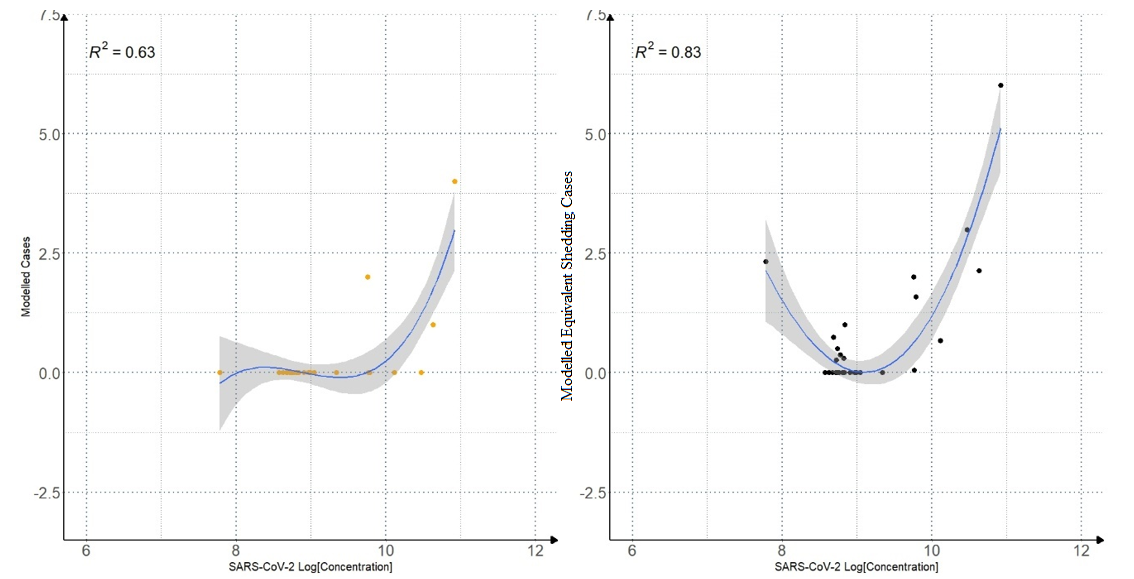

Supplement: Supplementary file 1 [file Data_Sheet_1.zip › Frontier_Epidemiology_English_EditingVersion_Figure16.docx]

Figure 17


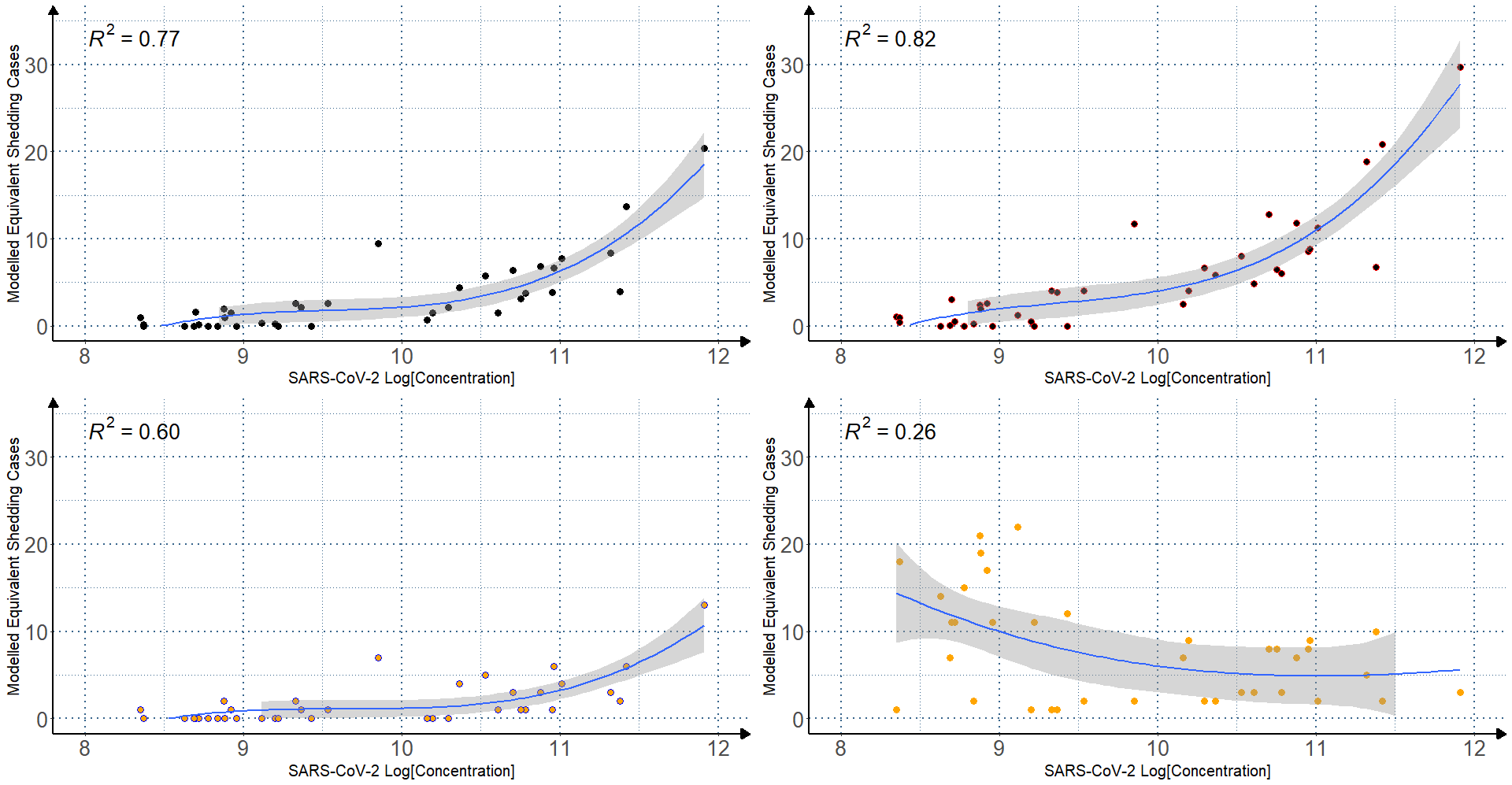

Supplement: Supplementary file 1 [file Data_Sheet_1.zip › Frontier_Epidemiology_SuppMat_Figure17.docx]

Figure 18


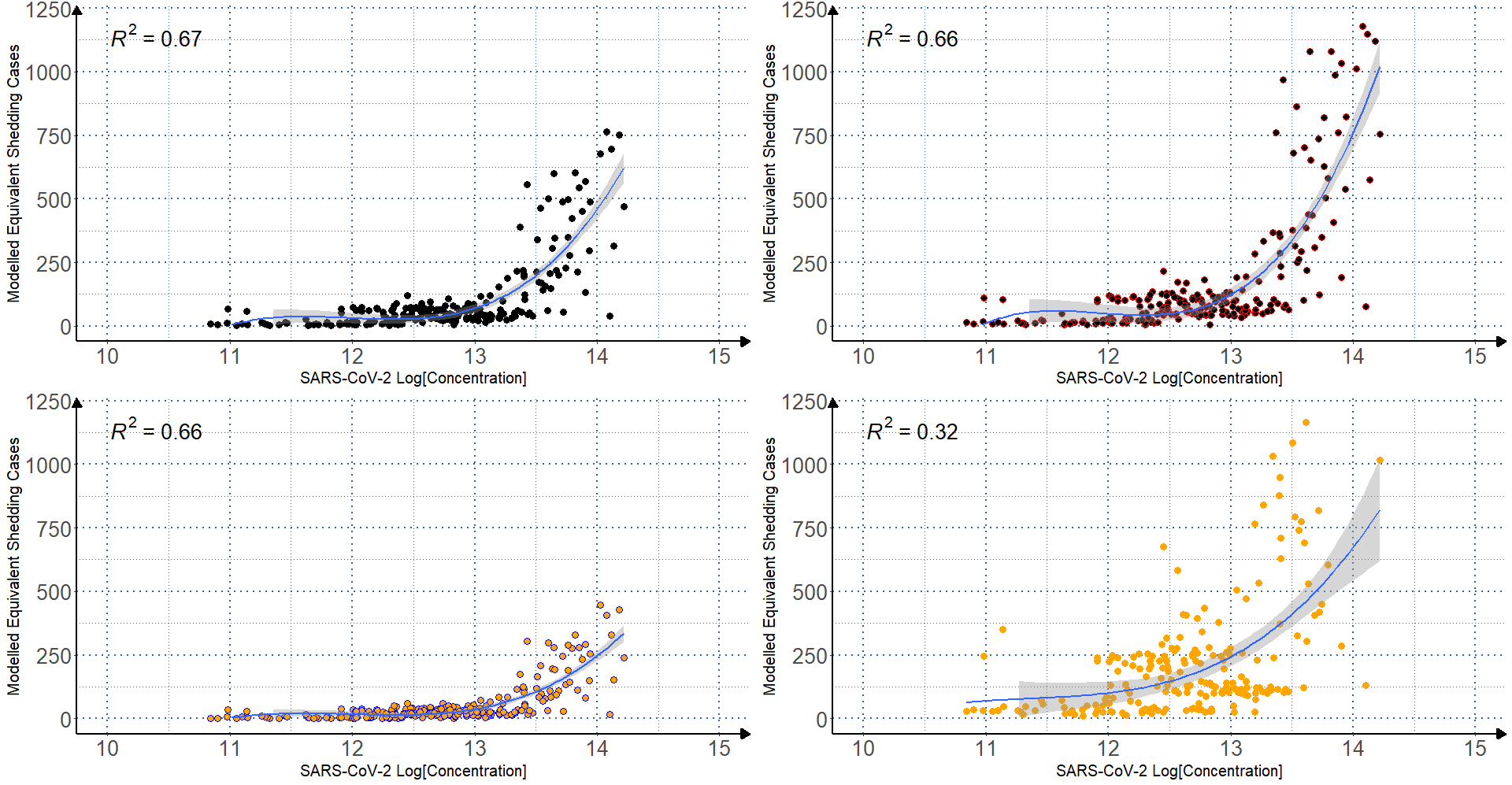

Supplement: Supplementary file 1 [file Data_Sheet_1.zip › Frontier_Epidemiology_SuppMat_Figure18.docx]

Figure 19


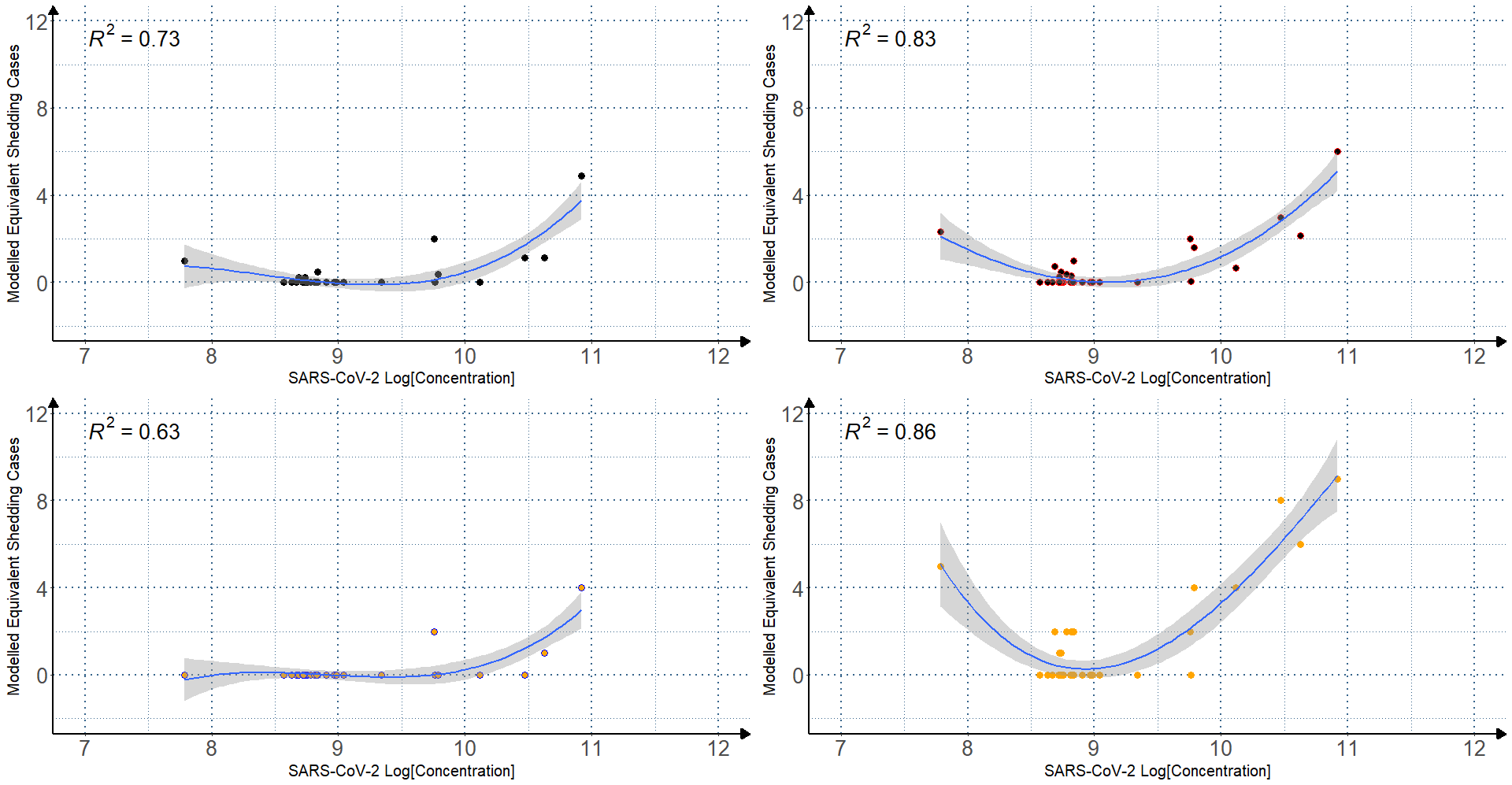

Supplement: Supplementary file 1 [file Data_Sheet_1.zip › Frontier_Epidemiology_SuppMat_Figure19.docx]

Figure 20


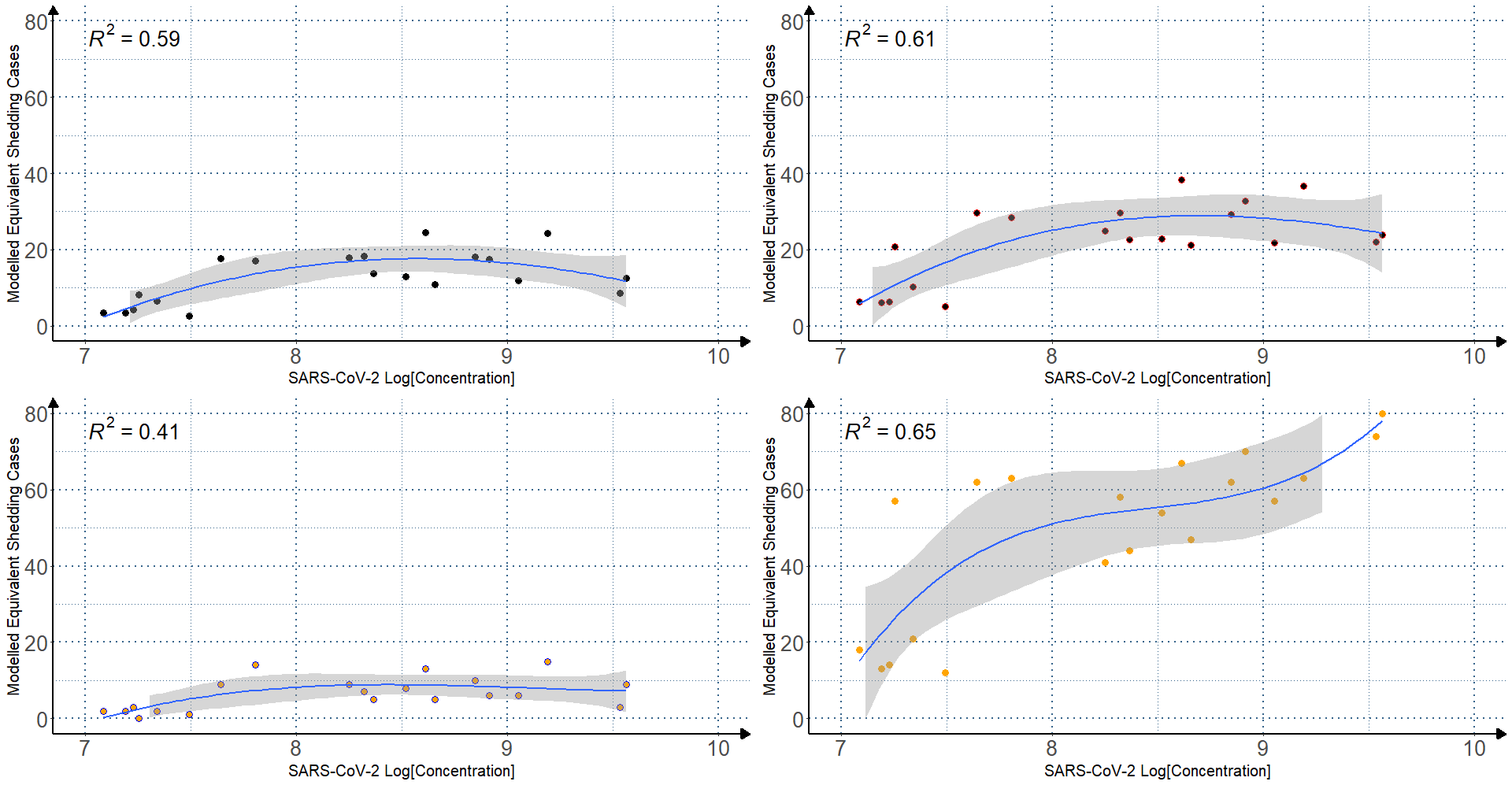

Supplement: Supplementary file 1 [file Data_Sheet_1.zip › Frontier_Epidemiology_SuppMat_Figure20.docx]

Figure 21


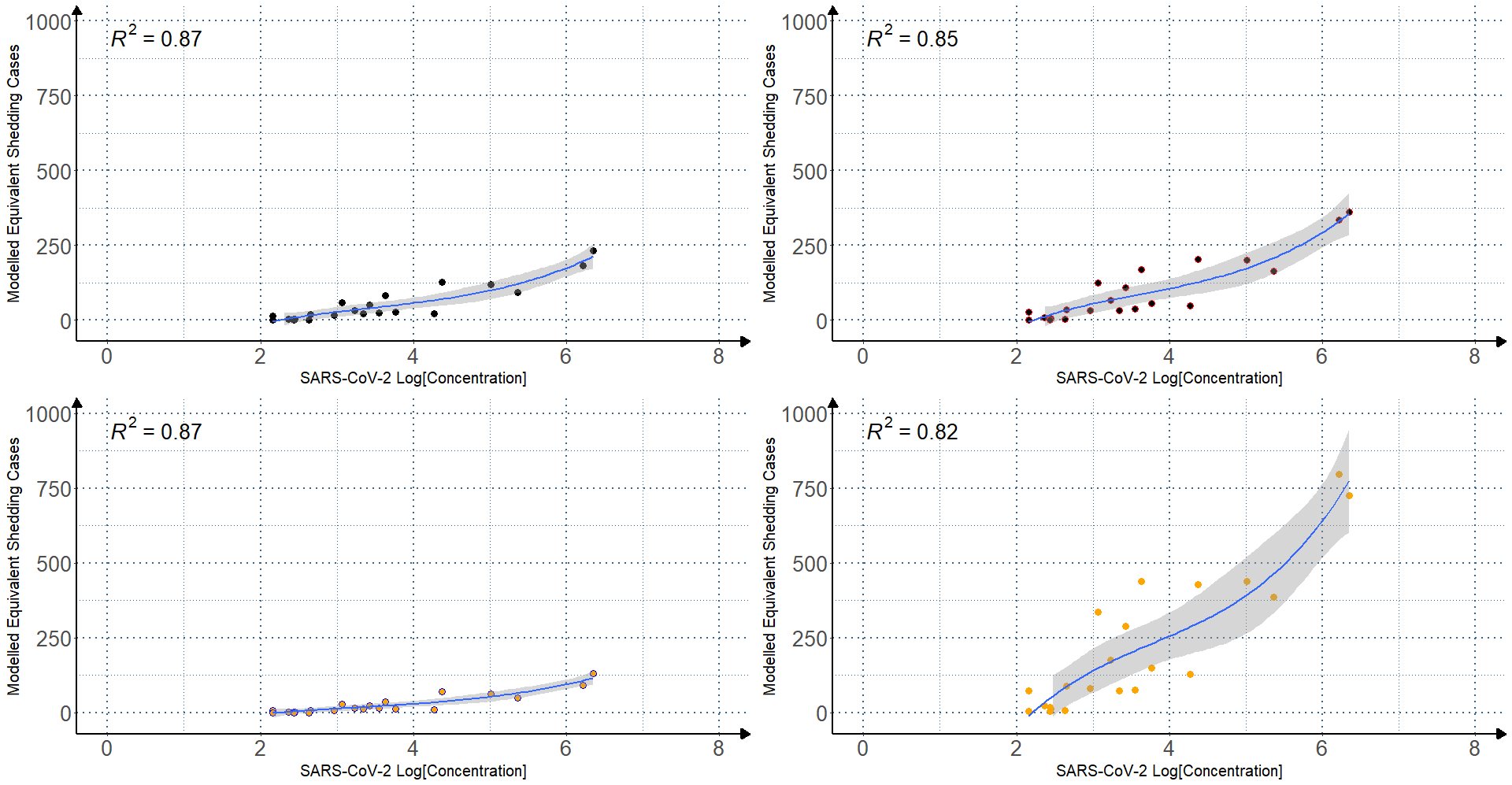

Supplement: Supplementary file 1 [file Data_Sheet_1.zip › Frontier_Epidemiology_SuppMat_Figure21.docx]

Figure 5


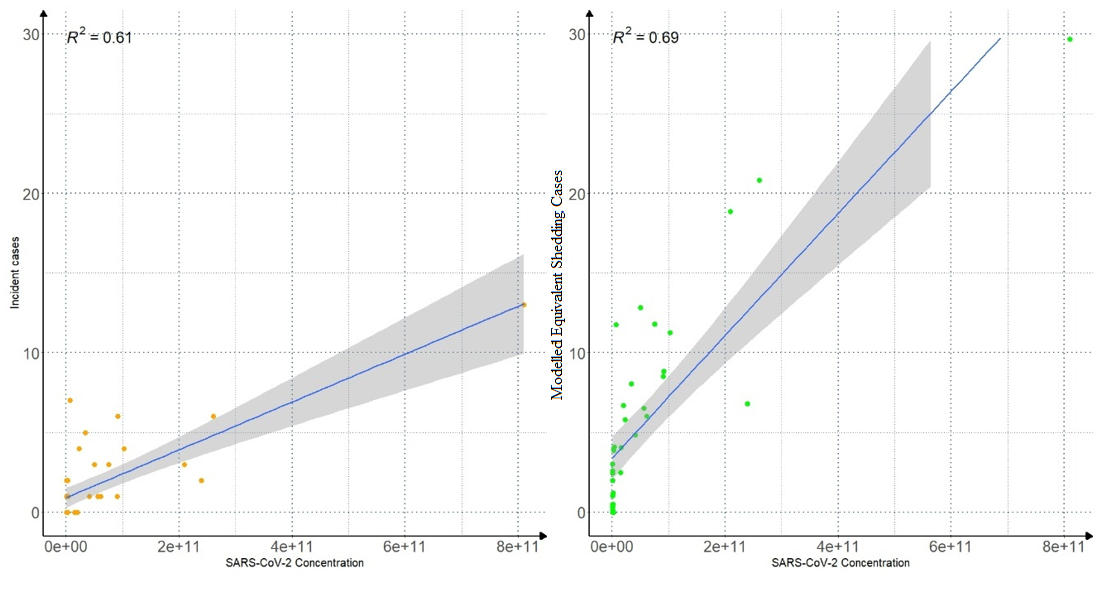

Supplement: Supplementary file 1 [file Data_Sheet_1.zip › Frontier_Epidemiology_English_EditingVersion_Figure5.docx]

Figure 6


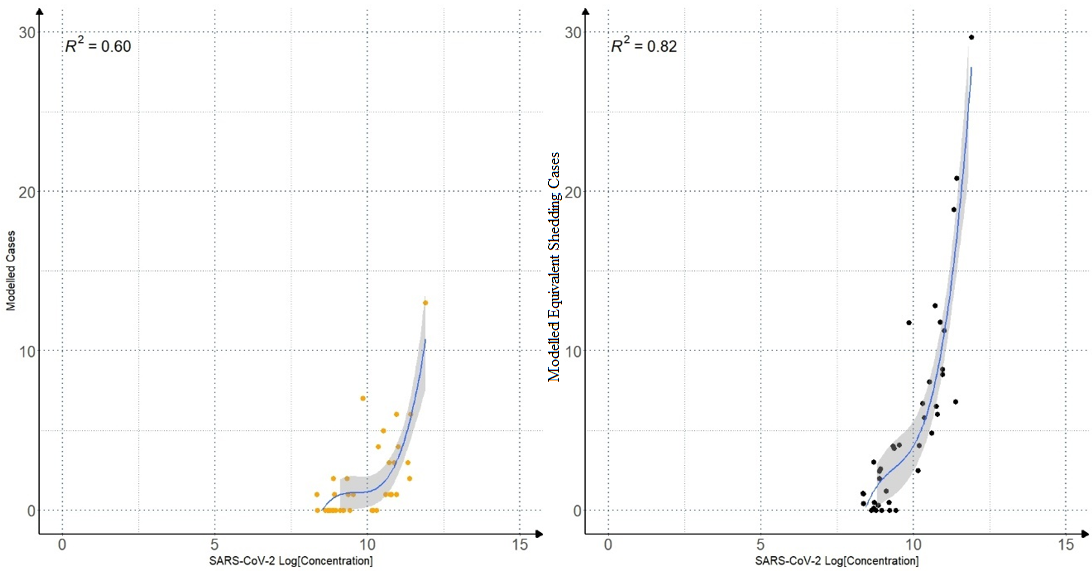

Supplement: Supplementary file 1 [file Data_Sheet_1.zip › Frontier_Epidemiology_English_EditingVersion_Figure6.docx]

Figure 7


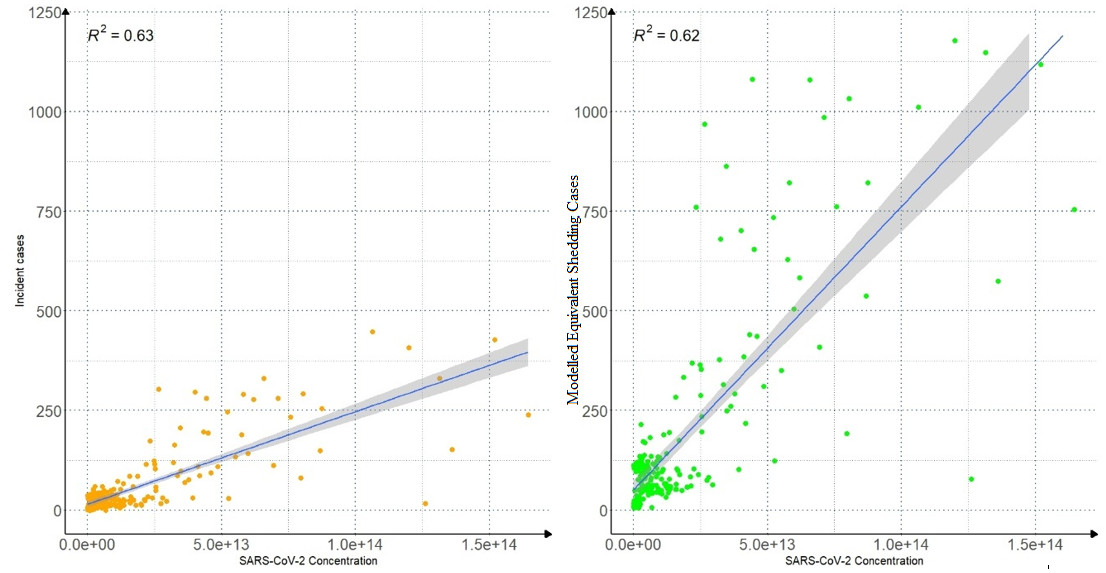

Supplement: Supplementary file 1 [file Data_Sheet_1.zip › Frontier_Epidemiology_English_EditingVersion_Figure7.docx]
